# Intrinsically Disordered Proteins and the Janus Challenge

**DOI:** 10.3390/biom8040179

**Published:** 2018-12-18

**Authors:** Prakash Kulkarni, Vladimir N. Uversky

**Affiliations:** 1 Department of Medical Oncology and Therapeutics Research, City of Hope National Medical Center, Duarte, CA 91010, USA; 2 Department of Molecular Medicine, Morsani College of Medicine, University of South Florida, Tampa, FL 33612, USA; 3 Laboratory of New methods in Biology, Institute for Biological Instrumentation, Russian Academy of Sciences, Pushchino 142290, Moscow Region, Russia

**Keywords:** intrinsically disordered protein, catalytic activity, conformational dynamics, origin of life theory

## Abstract

To gain a new insight into the role of proteins in the origin of life on Earth, we present the Janus Challenge: identify an intrinsically disordered protein (IDP), naturally occurring or synthetic, that has catalytic activity. For example, such a catalytic IDP may perform condensation reactions to catalyze a peptide bond or a phosphodiester bond formation utilizing natural/un-natural amino acids or nucleotides, respectively. The IDP may also have autocatalytic, de novo synthesis, or self-replicative activity. Meeting this challenge may not only shed new light and provide an alternative to the RNA world hypothesis, but it may also serve as an impetus for technological advances with important biomedical applications.

## 1. Presentation of the Janus Challenge

There are two fundamental questions in nature: What is life and how did it originate on the Earth? Although theories abound [1], including an extraterrestrial origin (panspermia) [2], questions regarding the origin and evolution of life remain rather elusive. Nonetheless, from an evolutionary perspective, it is generally accepted that RNA was the first biomolecule (the RNA-world hypothesis) [3]. The discovery that some RNAs have catalytic activity, coupled with the fact that RNA can store and replicate genetic information, bolstered this hypothesis. This idea also provided a conceptual framework for the existence of a prebiotic form that preceded the Last Universal Common Ancestor (LUCA), which had DNA as the genetic material, and from which all organisms now living on Earth that have a common descent. Finally, the discovery of reverse transcriptase and the enunciation of the central dogma of molecular biology where “DNA makes RNA and RNA makes protein” [4], further strengthened this concept. As a result, the role of proteins in the origin and evolution of life was relegated to the wayside. However, more recently, with renewed interest in the Foldamer hypothesis, proposed by Ken Dill, which accounts for how random chains could grow more informational and become autocatalytic (i.e., increasing their own concentrations), the role of proteins in evolution has once again come to the fore [5].

It is important to acknowledge that extensive fundamental molecular and biological evolution had to take place between the prebiotic origin of life and the LUCA. Tibor Gánti’s chemoton—an abstract model of the fundamental unit of life as a membrane-embedded autocatalytic subsystem comprising metabolism and replication process—represents one of the stages of prebiotic form [6]. Considering the evolutionary innovations and the origin of order between the pre-chemoton/chemoton and the LUCA from a self-organizing perspective, it is probable that short and unstructured peptides inclusive of unnatural amino acids, which are the prototypes of today’s intrinsically disordered proteins (IDPs), formed important constituents of the evolvable interacting networks. The circumspection of the prototypic IDPs and other biomolecules, including primitive ribonucleoproteins that formed interacting networks, into discrete units using membranous structures, could have led to the emergence of a chemoton-like unit, ultimately giving rise to the LUCA. However, empirical evidence demonstrating (a) the emergence of short IDPs using a template-independent synthesis mechanism to grow more informational and (b) the capability of IDPs to possess catalytic activity (including autocatalysis), is currently lacking.

We therefore present the Janus Challenge, which strives to identify an IDP, naturally occurring or synthetic, that has catalytic activity. For example, such a catalytic IDP can perform condensation reactions to catalyze a peptide or phosphodiester bond formation, utilizing natural/un-natural amino acids or nucleotides, respectively. The IDP may also have autocatalytic, de novo synthesis, or self-replicative activity. While there is no upper limit on the length of the IDP, it should be at least 30 amino acids long and >90% disordered, as determined experimentally.

Several observations inspired us to put out the Janus Challenge. First, given the vast phase space, even with limited rotational freedom of the dihedral angles, sporadic natural creation of a protein with a unique 3D structure is virtually improbable. Perhaps, it may have been easier for Nature to evolve a short helical peptide to perform catalytic activities with relatively high efficiency than randomly creating IDPs with poor catalytic capabilities. Given the natural propensity of helices for many of the amino acids, this may have been a possibility in the ancient world. However, as has been inferred, many of these amino acids, especially the non-polar aromatic ones, arrived much later during evolution than did the polar amino acids that characterize IDPs; in fact, the Miller–Urey experiment yielded only about half of the modern amino acids [7,8], suggesting that the genetic code evolved from a simpler form that encoded fewer amino acids [9], likely paralleled by the invention of biosynthetic pathways for new and chemically more complex amino acids [10]. Therefore, the temporal order of addition for the amino acids was proposed to be as follows: G/A, V/D, P, S, E/L, T, R, N, K, Q, I, C, H, F, M, Y, and W [11,12,13], rendering the latter possibility quite untenable. Nonetheless, with current advances in computational and experimental techniques, the possibility of identifying or designing a catalytic IDP seems realizable. Second, there are several examples of de novo and non-ribosomal synthesis of peptides that are biologically active, especially in the microbial world [14,15,16]. Third, there is precedence for polynucleotide chain elongation that is template-independent. For example, polyadenylation of messenger RNAs (mRNAs) at the 3′ end by poly(A) polymerase [17,18] and template-independent RNA synthesis by Qβ replicase [19,20]. Fourth, prion proteins can auto-replicate or propagate their functional or pathological state from one molecule to another. Fifth, the molten globule states of some enzymes have activity [21,22,23,24,25,26,27]. Finally, IDPs can interact with each other with picomolar affinities while fully retaining their structural disorder, long-range flexibility, and highly dynamic character [28].

The Romans considered Janus as the God of the beginning, transition, duality, and ending. He is usually depicted as having two faces, since he can look into the future and in the past. IDPs very likely, preceded the origin of life itself, transitioned to highly ordered proteins and, continued to exuberate as evolution progressed and more complex forms of life arose on the Earth. Furthermore, due to their chaotic conformational dynamics, they neither converge to a steady-state ensemble nor diverge to infinity. Thus, in many ways, the IDPs are an embodiment of the order/disorder duality in Nature, hence the Janus metaphor.

We trust that meeting this challenge will not only shed new light on the origin of life theory and even provide an alternative to the RNA world hypothesis, but may also serve as an impetus for technological advances. Lastly, such a breakthrough could have potential applications in biotechnology and medicine. As a reward, we offer to publish the paper in the journal, *Biomolecules,* free of charge.

## 2. Sciforum Discussion Group

In order to support the Janus Challenge and improve the communication within the IDP community, we have opened the discussion group, “Intrinsically Disordered Proteins and the Janus Challenge”, which is publicly available at the Sciforum site: https://sciforum.net/discussiongroup/display/IDPs_and_the_janus_challenge.

Therewith, we aim to set in motion a debate in which every scientist can share their interesting ideas and points of views regarding the science behind the Janus Challenge.

## 3. Selection Criteria of the Award

The award of the Janus Challenge consists in a fee waiver to publish the manuscript reporting results that meet this challenge in the journal, *Biomolecules.* The award will be given to the first article identifying an IDP (naturally occurring or synthetic) that has catalytic activity, only if this article is submitted to and published after successful peer review and processing in the journal, *Biomolecules*.

In order to be eligible for the award, the candidate’s work must comply with the following terms:

- The work needs to meet the standards to be considered as an original research article. Other types of papers (e.g., short communications, commentaries, hypothesis, etc.) will be excluded from the selection process.
- The article needs to be submitted to *Biomolecules* using the online submission system. The authors will have to include a cover letter indicating that their work aims to be considered for the Janus Challenge Award. A pre-submission may be sent to the *Biomolecules* Editorial Office or to the corresponding authors of this Editorial.
- The paper will be submitted to peer-review, which will meet the requirements established by the journal *Biomolecules*.
- The ‘first position’ will be defined on the basis of the submission date of the manuscript reporting the aforementioned results to the journal *Biomolecules*. If the manuscript is not accepted for publication or is withdrawn, the position ‘first’ will remain available for future submission.
- The IDP or the protein containing intrinsically disordered regions with catalytic activity should have been obtained experimentally, either isolated or synthetized. Theoretical but not experimentally confirmed proteins are not eligible for the award.
- The ‘active site’ (region where the substrate bind and undergo the chemical reaction) of such a primordial protein must be disordered or must have a transient ‘structure’ at the best.

The Academic Editors and the Editorial Office of the journal *Biomolecules* reserve the right to determine if a manuscript is suitable for the award.
